# Microvascular endothelial dysfunction is associated with albuminuria and CKD in older adults

**DOI:** 10.1186/s12882-016-0303-x

**Published:** 2016-07-13

**Authors:** Stephen L. Seliger, Shabnam Salimi, Valerie Pierre, Jamie Giffuni, Leslie Katzel, Afshin Parsa

**Affiliations:** Department of Medicine, University of Maryland School of Medicine, 22 S. Greene Street, Room N3W143, Baltimore, MD 21201 USA; Medicine, VA Maryland Healthcare System, Baltimore, USA; Epidemiology and Public Health, University of Maryland School of Medicine, Baltimore, USA; Creighton University School of Medicine, Omaha, USA; GRECC, VA Maryland Healthcare System, Baltimore, USA

**Keywords:** Endothelial function, CKD, Albuminuria, Vascular disease, Blood pressure, Laser Doppler flowmetry

## Abstract

**Background:**

Impairment in glomerular endothelial function likely plays a major role in the development of albuminuria and CKD progression. Glomerular endothelial dysfunction may reflect systemic microvascular dysfunction, accounting in part for the greater cardiovascular risk in patients with albuminuria. Prior studies of vascular function in CKD have focused on conduit artery function or those with ESRD, and have not examined microvascular endothelial function with albuminuria.

**Methods:**

We conducted a cross-sectional study among older hypertensive male veterans with stage 1–4 CKD, and hypertensive controls without CKD. Microvascular function was quantified by two distinct Laser-Doppler flowmetry (LDF) measures: peak responses to 1) post-occlusive reactive hyperemia (PORH) and 2) thermal hyperemia (TH), measured on forearm skin. Associations of each LDF measure with albuminuria, eGFR, and CKD status were estimated using correlation coefficients and multiple linear regression, accounting for potential confounders.

**Results:**

Among 66 participants (mean age 69.2 years), 36 had CKD (mean eGFR 46.1 cc/min/1.73 m^2^; 30.6 % with overt albuminuria). LDF responses to PORH and TH were 43 and 39 % significantly lower in multivariate analyses among those with macroalbuminuria compared to normoalbuminuria, (β= − 0.42, *p* = 0.009 and β= −0.37, *p* = 0.01, respectively). Those with CKD had a 23.9 % lower response to PORH compared to controls (*p* = 0.02 after adjustment). In contrast, TH responses did not differ between those with and without CKD.

**Conclusions:**

Microvascular endothelial function was strongly associated with greater albuminuria and CKD, independent of diabetes and blood pressure. These findings may explain in part the excess systemic cardiovascular risk associated with albuminuria and CKD.

## Background

The risk of cardiovascular related morbidity and mortality in individuals with chronic kidney disease (CKD) is exceedingly high and often greater than the risk of progressing to end-stage renal disease (ESRD), especially in older adults [1]. However, the mechanisms which account for the systematic vascular manifestations of albuminuria and CKD are not fully elucidated. The endothelium, a single cell layer providing the interface between circulating blood and the vascular wall, regulates many critical aspects of vascular function including vascular tone, coagulation, and protection against oxidative stress and inflammation [2]. It has been proposed that elevated urine albumin excretion-especially low to moderate elevations - represents a state of generalized endothelial dysfunction, but there has been relatively limited direct evidence in humans to support this hypothesis [3]. Moreover, dysfunction of the endothelium is increasingly recognized as a sentinel event in the development and progression of both focal and systemic vascular disease. Indeed, markers and measures of endothelial dysfunction, a determinant of systemic vasculopathy, have been shown to be increased in CKD [4]. However, a variety of endothelial function measures have been employed, each with advantages and weaknesses. Most prior studies examining endothelial function have focused on ischemic flow mediated dilatation (FMD) of medium sized conduit arteries such as the brachial artery. These studies have demonstrated a significant impairment in the endothelium-dependent vasodilatation of the brachial artery among patients with ESRD, late stage CKD, or patients with glomerulonephritis, compared to controls [5–9]. Although such conduit artery function represents an important potential mediator of vascular risk, the renal vascular bed consists predominately of micro-vessels. Therefore, dysfunction of the microvascular endothelium may better characterize the systemic vasculopathy of CKD [10, 11]. Studies of endothelial function and albuminuria have been less consistent, in the context of variable methodological approaches and populations and have not focused on albuminuria in patients with HTN with and without moderate CKD [8, 12–15]. Non-invasive evaluation of the cutaneous microcirculatory is readily feasible using the technique of Laser- Doppler flowmetry (LDF). Specifically, the semi-automated use of LDF to measure cutaneous microcirculatory function in response to specific provocations has been advocated as a practical and reliable in-vivo assay of systemic microvascular vasodilatory function [16, 17]. A small number of prior studies using LDF techniques have demonstrated deficits in endothelium-dependent microvascular function in patients with ESRD and advanced non-dialysis dependent CKD compared to controls, and an association of these functional measures with cardiovascular risk [10, 18–20]. However, it is unclear whether microvascular endothelial dysfunction is present among those with earlier stages of CKD, and whether common comorbidities in those with CKD such as hypertension and diabetes account for this dysfunction.

We therefore performed an observational, cross-sectional study to examine the relationship of microvascular endothelial function with non-dialysis CKD and measures of renal disease severity (albuminuria and eGFR) among older adults with treated hypertension. We hypothesized that those older adults with greater albuminuria and lower eGFR would have more diminished endothelial function as assessed by two separate laser Doppler flowmetry based measures, after accounting for demographic and co-morbid factors.

## Methods

### Study subjects

Community-dwelling hypertensive outpatients at the VA Maryland Healthcare System aged 60 or older with and without chronic kidney disease (stages 1–4) or diabetes mellitus were eligible for participation. CKD was defined according to K/DOQI criteria as persistent eGFR < 60 cc/min/1.73 m^2^ or abnormal albuminuria for at least 3 months. Patients with any of the following conditions were excluded from participation: NYHA class III-IV heart failure; coronary revascularization or acute coronary syndrome within the prior 3 months; vascular claudication; prior stroke; dementia; severe anemia (Hb < 9 g/dL), and poorly controlled hypertension (BP > 180/95) or diabetes (HbA1C > 11 %). Participants were recruited from nephrology, diabetes, and primary care clinics. Participants were enrolled consecutively from 11/2010 through 2/2013, without specific matching of CKD and non-CKD patients.

### Covariates and measurements

Clinical co-morbidity was determined through review of the electronic medical record and participant interview. Coronary artery disease was defined as a history of prior myocardial infarction, coronary revascularization, and/or coronary disease by angiography. Albuminuria was quantified with the urine albumin/creatinine ratio on a spot morning urine sample after an overnight fast. Albuminuria was classified as per standard criteria as normoalbuminuria if albumin/creatinine ratio < 30 mg/gm; microabluminuria if albumin/creatinine ratio was 30 to 300 mg/gm and macroalbuminuria if albumin/creatinine ratio was > 300 mg/gm. Blood pressure was recorded in the sitting position prior to LDF measurements using a Critikon Dinamap 1846 SX monitor (GE Medical Systems), and mean arterial pressure calculated as (DBP + (SBP-DBP)/3). GFR was estimated from serum creatinine measured with an IDMS-traceable assay using the 4-variable MDRD equation.

### Laser Doppler flowmetry (LDF)

LDF uses a laser light source and Doppler shift effect to non-invasively measure microvascular blood flow on a relative BPU (Blood Perfusion Units) scale. This allows for the measurement of relative change in microcirculatory perfusion in response to various interventions. All measurements were performed according to a standardized protocol with a Perimed, PF5020 unit in a temperature-controlled room after an overnight fast and after withholding of AM doses of vasoactive medications including nitrates. Active smokers were instructed to abstain from smoking the day of LDF testing. We used two distinct LDF based measures: 1) post occlusive reactive hyperemic (PORH) and 2) thermal hyperemic (TH) mediated response. In TH-LDF, the LDF probe is placed on the volar aspect of the forearm and heated to 44C causing dilatation of the skin vessels and increased flow, which is measured over 30 min. TH-LDF results in a biphasic response, an initial axon reflex peak phase which is mediated in part by the neuropeptides substance P and calcitonin gene-related peptide (CGRP). This is followed by a more prolonged plateau phase, largely mediated by the endothelial release of nitric oxide [21]. For PORH-LDF, the upper arm flow is occluded by a blood pressure cuff for a 4-min period. The resultant ischemia induces dilatation of resistance vessels. Upon release of the cuff, there is a rush of blood flow secondary to the induced decreased arterial resistance, which in turn causes increase shear stress on the vessels, inducing further dilatation by endothelial and potentially non-endothelial based mediators. All blood flow tracings were digitally transferred and interpreted by a single reviewer (AS) blinded to clinical characteristics of the participant. Tracings with technical deficiencies precluding accurate quantification (eg participant motion, displacement of probe during recording) were excluded from analysis. The percent increase in flow from baseline is then used as a measure of endothelial function and vascular reactivity for both TH and PORH-LDF measures [21]. Additionally, PORH- and TH-LDF were categorized as high or low, if above or below the median for each measure, respectively.

### Statistical methods

Clinical characteristics between those with and without CKD were compared using the independent-sample *t*-tests for continuous variables and chi-squared or Fisher’s exact test for categorical variables. Comparisons of each LDF measure across albuminuria category were performed with ANOVA tests. Correlations of each LDF measure (response to TH and PORH) with albuminuria were estimated using Pearson correlation coefficients, after natural logarithm (ln- transformation) of the urine albumin/creatinine ratio to ensure a normal distribution. The association of albuminuria and CKD with each LDF measure (dependent variable) after adjusting for demographic and co-morbid factors was estimated using multiple linear regression. Adjustment covariates were selected a priori and included age, self-reported race (African-American vs. other), mean arterial pressure, coronary artery disease, and diabetes. Associations were presented as standardized regression coefficients ($\widehat{\upbeta}$) to allow comparison across different outcome measurements. The Cook-Weisberg test was used to confirm the assumption of homoscedasticity.

## Results

Cutaneous PORH and/or TH provocations measures were obtained on 66 male participants. Of these, 61 PORH and 52 TH tracing were determined to be of sufficient technical quality for quantification of peak response. Compared to those without CKD (*N* = 30), those with CKD (*N* = 36) were overall well matched with similar age, race, blood pressure, body mass index, use of renin-angiotensin antagonists, and smoking history, but were more likely to have diabetes (*p* < 0.01) (Table 1.) Of those with CKD, 11 (30.6 %) had overt albuminuria, with mean (SD) eGFR_MDRD_ 46.1 (2.4) cc/min/1.73 m^2^; 4 (11 %) had stage 2, 28 (78 %) had stage 3, and 4 (11 %) had stage 4 CKD.

**Table 1:** Baseline characteristics of study sample

	No CKD (*N* = 30)	CKD (*N* = 36)	*p*-value
Age	68.6 (1.4)	69.7 (1.2)	0.5
African American	18 (60 %)	18 (50 %)	0.7
SBP (mmHg)	137.6 (19.2)	143.1 (16.6)	0.2
DBP (mmHg)	74.0 (10.5)	74.8 (8.7)	0.7
BMI	30.4 (5.5)	31.0 (6.3)	0.7
Diabetes	6 (20 %)	27 (75.0 %)	<0.01
Coronary Artery Disease^a^	2 (6.7 %)	8 (22.2 %)	0.1
Smoking			
Current	0 (0 %)	1 (2.8 %)	0.5
Former	21 (70 %)	21 (58.3 %)	
Never	9 (30 %)	13 (36 %)	
ACEI or ARB	20 (67 %)	28 (77.8 %)	0.3
Nitrates	0	2	0.3
eGFR (cc/min/1.73 m2)	85.4 (19.2)	46.1 (2.4)	
Alb/Cr ratio	3.8 [2.9, 7.8]	83.3 [16.6, 367.0]	

Cell values represent mean (SD), median [inter-quartile range], and N(%)

^a^Patients with unstable coronary disease or with recent coronary intervention were excluded

### PORH-LDF

**Fig. 1:**
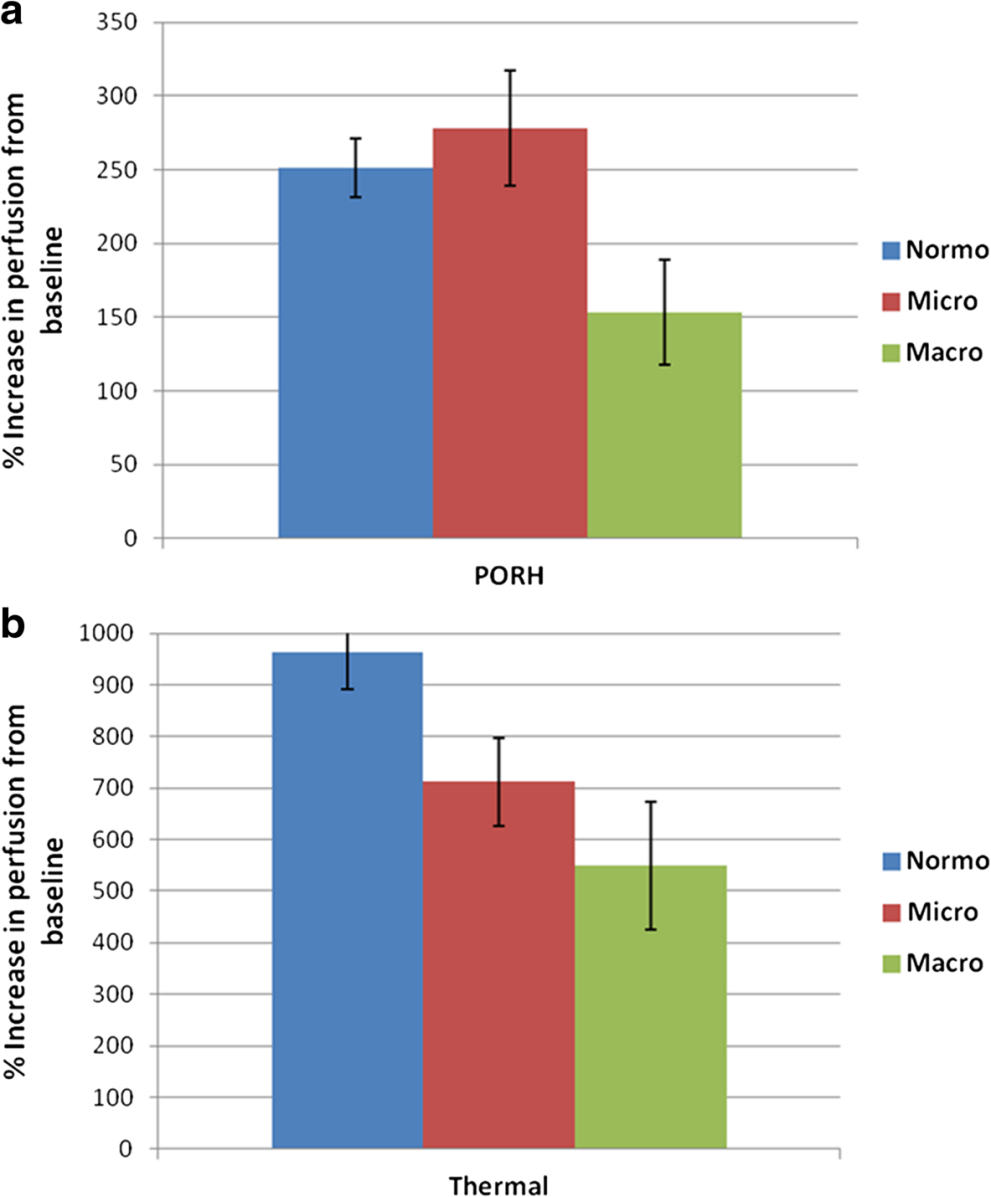
Hyperemic response to post-occlusive (**a**) and thermal (**b**) provocations, by albuminuria category

**Fig. 2:**
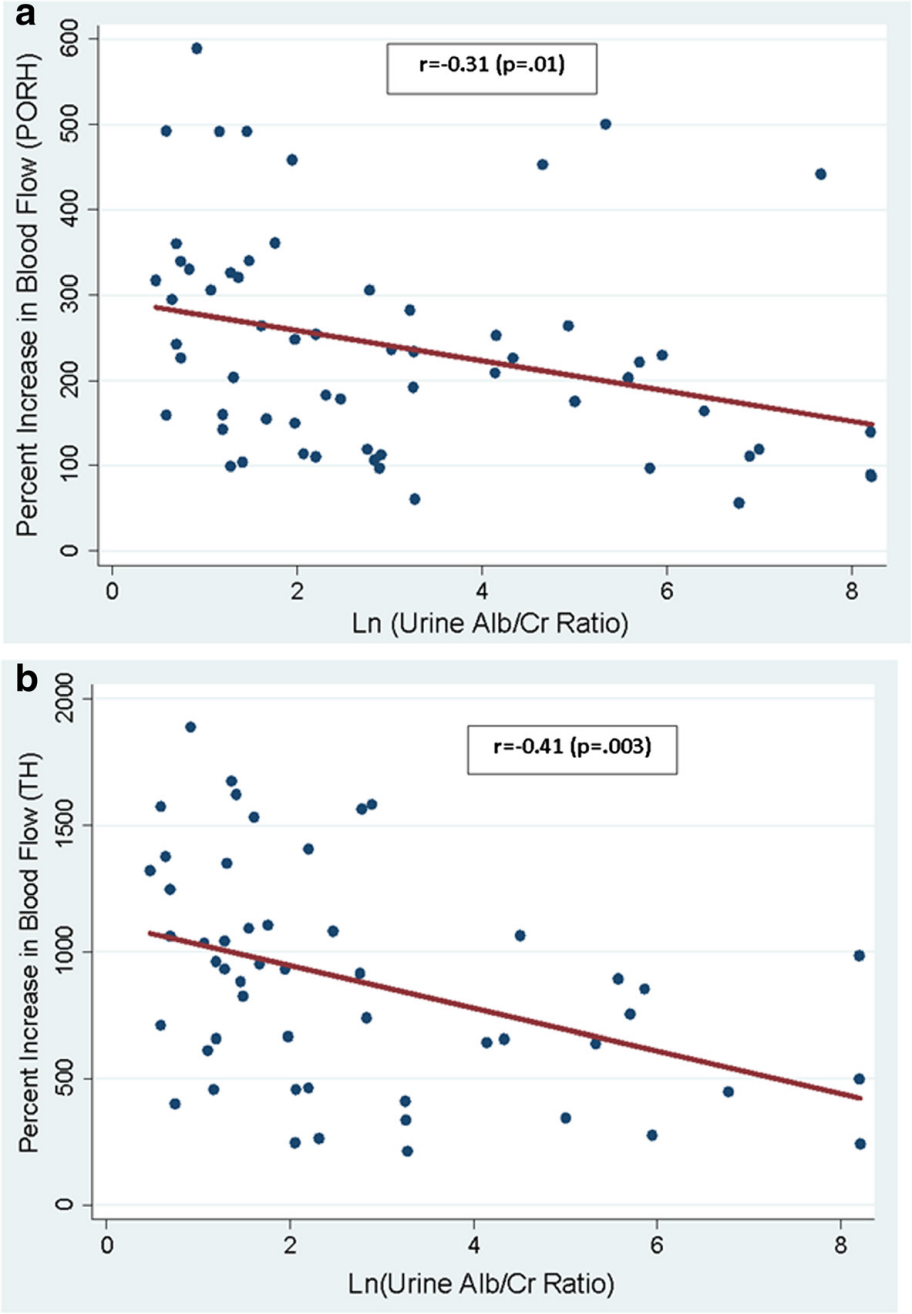
Association of hyperemic response to post-occlusive (**a**) and (**b**) thermal provocations with albuminuria

**Fig. 3:**
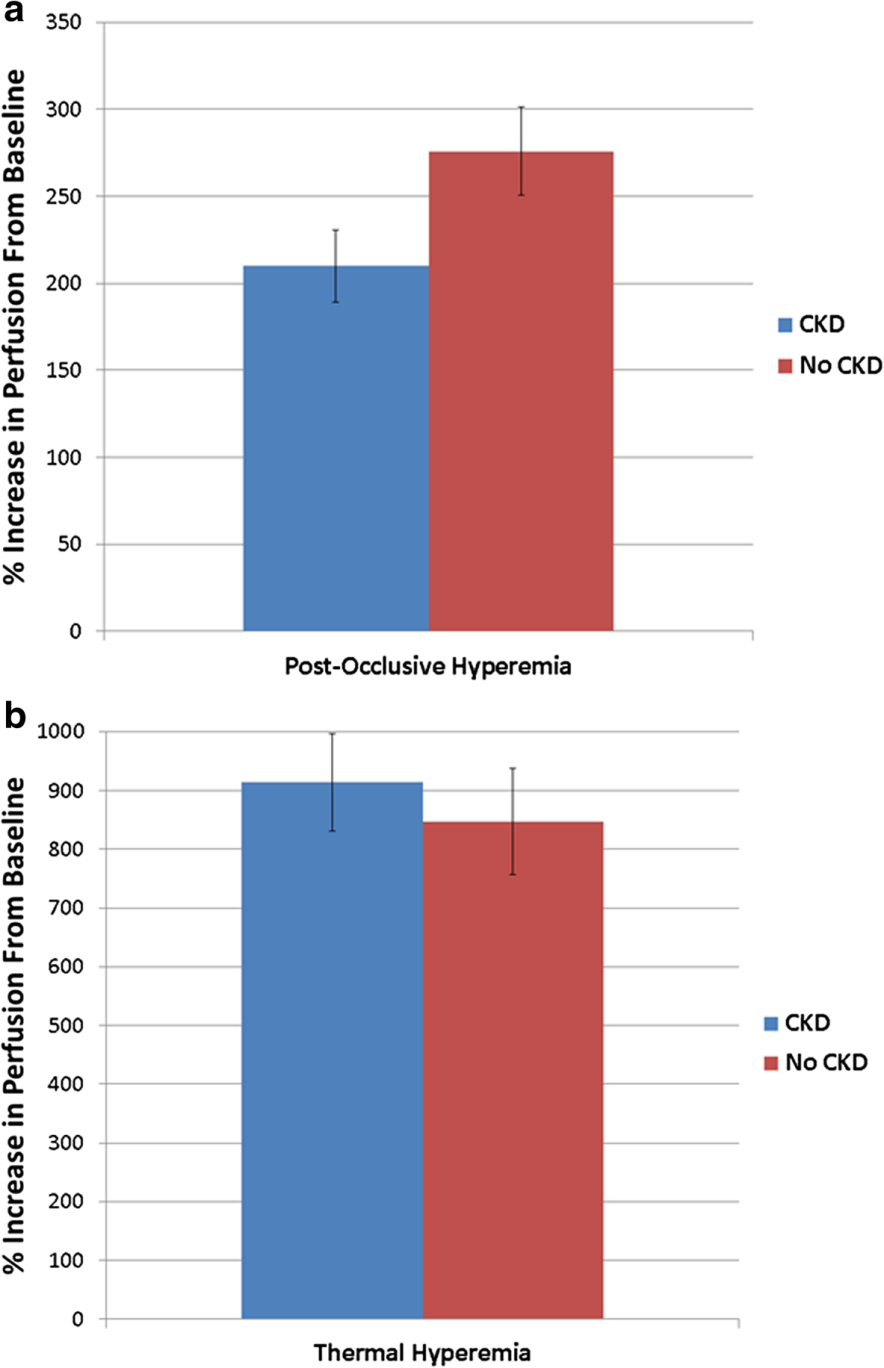
Hyperemic response to post-occlusive (**a**) and thermal (**b**) provocations, by albuminuria category, among those with and without CKD

Comparing those with high vs. low PORH-LDF, those with lower post-occlusive microvascular reactivity had modestly lower systolic blood pressure and significantly lower eGFR and higher albuminuria (Table 2.) In univariate analysis, PORH-LDF was 43 % lower among those with macroalbuminuria compared to those with normoalbuminuria (Fig. 1a; 95 % CI = 0.10 to 0.90 %, *p* = 0.03) and was inversely correlated with albuminuria (r = −0.31, *p* = 0.01) (Fig. 2a). After adjustment for demographic factors, blood pressure and diabetes, PORH-LDF remained inversely associated with greater albuminuria (Table 3; $\widehat{\upbeta}$= − 0.37, 95 % CI = −0.55 to −0.08, *p* = 0.01.) The association between PORH and albuminuria did not differ with diabetic status (all tests of interaction, *p* > 0.5). eGFR as a continuous variable was not associated with PORH-LDF measures (*p* = 0.2.) Among those with CKD, PORH-LDF response was 24%lower compared to non-CKD controls (Fig. 3a: 95 % CI = −0.61 % to −0.05 %, *p* = 0.045). After multivariate adjustment, this association remained significant (Table 3; $\widehat{\upbeta}$= − 0.38, 95 % CI = −0.62 to −0.01, *p* = 0.02).

**Table 2:** Characteristics of study sample, by Post-Occlusive and Thermal Hyperemic categories

	Post-Occlusive Reactive Hyperemia(*N* = 65)			Thermal Hyperemia (*N* = 52)		
	High	Low	p-value	High (>892 %)	Low (<892 %)	p-value
Age	68.6	70.6 (7.6)	0.3	68.5 (7.3)	69.8 (1.5)	0.5
African American	15 (48.4 %)	17 (56.7 %)	0.5	17 (65.4 %)	12 (46.2 %)	0.2
SBP (mmHg)	145.5 (15.0)	136.0 (18.3)	0.03	141.3 (16.0)	137.2 (18.8)	0.4
DBP (mmHg)	76.5 (10.0)	72.1 (8.1)	0.06	75.4 (10.2)	73.2 (8.1)	0.4
BMI	30.7 (6.6)	30.4 (5.5)	0.9	30.2 (6.3)	32.6 (4.8)	0.14
Diabetes	13 (41.9 %)	18 (60 %)	0.2	16 (61.5 %)	10 (38.5 %)	0.10
Coronary Artery Disease^a^	5 (16.1 %)	5 (16.7 %)	1.0	4 (15.4 %)	4 (15.4 %)	1.0
Smoking						0.6
Current	19 (61.3 %)	19 (63.3 %)	0.6	1 (3.9 %)	0 (0 %)	
Former	0 (0 %)	1 (3.3 %)		15 (57.7 %)	17 (65.4 %)	
				10 (38.5 %)	9 (34.6 %)	
Never	12 (38.7 %)	9(30 %)				
ACEI or ARB	19 (63.3 %)	26 (83.9 %)	0.07	18 (69 %)	18 (69 %)	1.0
Nitrates	1 (3.3 %)	1 (3.3 %)	0.8	1 (3.9 %)	0	0.5
eGFR (cc/min/1.73 m2)	69.2 (4.9)	56.0 (4.1)	0.04	61.9 (24.0)	68.7 (29.8)	0.4
Alb/Cr ratio	5 [2.3, 26]	18.2	0.006	4.4	21.5	0.009
		[7.2, 334]		[2.8, 11.8]	[4.4, 207]	

Cell values represent mean (SD), median [inter-quartile range], and N (%)

^a^Patients with unstable coronary disease or with recent coronary intervention were excluded

**Table 3:** Association ($\widehat{\upbeta}$) of microvascular endothelial function with CKD, albuminuria, and eGFR

	Post-Occlusive Hyperemia (*N* = 61)		Thermal Hyperemia (*N* = 52)	
	Unadjusted	Adjusted	Unadjusted	Adjusted
Albuminuria	−0.31 (*p* = 0.01)	−0.37 (*p* = 0.01)	−0.41 (*p* = 0.003)	−0.42 (*p* = 0.009)
CKD (vs. no CKD control)	−0.25 (*p* = −0.045)	−0.38 (*p* = 0.02)	−0.08 (*p* = 0.6)	0.09 (*p* = 0.6)
eGFR	0.17 (*p* = 0.2)	0.20 (*p* = 0.2)	0.12 (*p* = 0.41)	−0.02 (*p* = 0.9)

Albuminuria: Natural log- transformed Urine albumin to creatinine ratio, Cell values represent standardized regression coefficient

Adjusted for age, race (African-American vs. other), mean arterial blood pressure, diabetes, and coronary artery disease

### TH-LDF

Consistent with our PORH-LDF based results, those with lower TH reactivity had higher albuminuria (Table 2; *p* = 0.009). Albuminuria was inversely and strongly correlated with TH-LDF (Fig. 2b:r = −0.41, *p* = 0.003.) TH-LDF measures were 39 % lower among those with macroalbuminuria compared to normoalbuminuria (Fig. 1b; *p* = 0.03.) After adjustment for demographic factors, and major co-morbidities, greater albuminuria was associated with lower TH-LDF measures (Table 3.) Of note, the association of TH-LDF measures with albuminuria ($\widehat{\upbeta}$= − 0.42) was markedly greater than the associations of age (β=0.15) and mean arterial pressure (β=0.08).

In contrast with PORH-LDF, there was no significant association of CKD or eGFR with TH-LDF based measures (Fig. 3b and Table 3; *p* = 0.6 and *p* = 0.9, respectively.)

The relationship between LDF responses with albuminuria did not differ between those with and without diabetes in either PORH- or TH-LDF (*p* > 0.5).

## Discussion

Among older hypertensive men with and without non-dialysis dependent CKD, microvascular endothelial function as estimated by the cutaneous hyperemic response to brachial artery occlusion and localized heating is decreased in a graded, dose-dependent fashion with greater albuminuria. Furthermore, those with CKD had a 24 % lower PORH response than those without CKD. These associations were robust to adjustment for selected potential confounders including blood pressure, and were qualitatively greater than the associations of other factors such as greater age and blood pressure.

Even low-level elevations of albuminuria are associated with measureable increases in risk of vascular disease, including myocardial infarction, stroke, cardiovascular death, and cognitive decline, especially in older adults. While podocyte function has long been recognized as a key component of the glomerular albumin barrier, there has been continued evidence demonstrating that not all proteinuria can be accounted for based on podocyte dysfunction, such as in early diabetic nephropathy and systemic inflammatory states (eg, sepsis and inflammatory bowel disease). Recent evidence strongly suggests a prominent role for the glomerular endothelial layer and associated glycocalyx as a key barrier to albumin across the glomerular basement membrane [22]. This finding would potentially account in part for the strong association between albuminuria and cardiovascular disease risk, independent of decreased GFR. The results of this study provide support for this hypothesis, suggesting that – at least in older adults with hypertension – microvascular endothelial function is a major correlate of albuminuria, independent of blood pressure, diabetes and decreased GFR.

Prior studies using LDF to measure microvascular function in CKD patients have primarily focused on those with ESRD [10, 11, 18, 20, 23], whether dialysis-dependent or recipients of renal transplants. These studies have generally reported significantly lower endothelial function in ESRD or advanced CKD patients [19] compared to controls, although there have been discrepant results regarding whether these differences are independent of hypertension [18]. Prior studies in ESRD have also suggested that these measures are prognostically important for short-term prediction of cardiovascular events in dialysis-dependent patients [11] and for allograft failure in renal transplant recipients [23]. Moreover, since various measures of endothelial function and vascular reactivity are only modestly correlated to each other as they capture various components of endothelial and vascular response [21], we used two methods, TH and PORH to better ascertain microvascular responses. This allowed us to estimate the association between CKD and PORH, which may have been missed by using only TH based measures, extending previous findings of reduced microvascular endothelial function to those with mild-moderate CKD. Lastly, by comparing CKD patients to age matched controls with hypertension, as opposed to healthy controls without any co-morbidity, our findings suggest that the endothelial functional impairment is not explained merely by the increased prevalence of hypertension in CKD or diabetes.

Previous studies of brachial FMD and/or LDF based measures of vascular and endothelial function have yielded conflicting results regarding associations with proteinuria. Most notably, many of these were in patients with diabetes and did not explore association between endothelial function measures and proteinuria, independent of diabetes status. Several involved patients with non-diabetic and non-hypertensive glomerulonephritis or patients with HIV, which cannot be generalized to the patient with hypertension-attributed CKD. Our findings of strong consistent dose dependent association between two distinct measures of microvascular endothelial function and reactivity, independent of blood pressure and diabetes, meaningfully extend previous findings. Moreover, while obtaining reliable brachial artery FMD measures can be technically challenging and costly [24], the semi-automated LDF based TH and PORH measures of skin microvascular reactivity require minimal training, are relatively easy to obtain and have good reproducibility and may at times be preferred over other non-invasive measures of endothelial function and vascular reactivity [25–27]. Furthermore, LDF measures are considered to be less dependent on systemic arterial blood pressure, as they reflect blood flow difference in the microcirculatory vessels, which are not subject to the high pressure in conduit vessels such as the brachial artery, and further represent difference in flow from the baseline, which would normalize any BP-related difference in baseline blood flow.

Limitations of the present study include the modest sample size, which precluded extensive subgroup analysis and limited the potential number of confounders that could be considered in multivariate models. Although those without CKD were of similar age, ethnic background, and blood pressure to those with CKD, they were considerably less likely to have diabetes and stable coronary heart disease. Although we adjusted for these differences in regression models, residual confounding may have persisted. The study population consisted of older men-consistent with the recruitment setting of a VA medical center – and the results may not apply to women or middle-aged adults. Due to the cross-sectional design of the study, we cannot determine whether microvascular dysfunction is a causal factor for albuminuria and CKD, or a result of the renal disease.

## Conclusions

In conclusion, we find that LDF-based measures of endothelial function and microvascular reactivity are the strongest determinants of albuminuria in our older hypertensive population of patients with and without CKD, irrespective of diabetes status. We also found our patients with CKD to have lower microvascular reactivity based on our PORH measures. These findings highlight that skin measures of microvasculatory endothelial function and reactivity as a proxy of systemic endothelial function are strongly correlated with albuminuria and that albuminuria may plausibly reflect glomerular microvasculatory dysfunction. This provides further evidence in favor of the hypothesis that endothelial function comprises a common pathophysiology linking renal dysfunction and albuminuria with systemic vascular disease.

## Abbreviations

CKD, chronic kidney disease; ESRD, end-stage renal disease; FMD, flow-mediated dilatation; LDF, laser Doppler flowmetry; PORH, post-occlusive reactive hyperemia; TH, thermal hyperemia
